# Antiviral Roles of Abscisic Acid in Plants

**DOI:** 10.3389/fpls.2017.01760

**Published:** 2017-10-11

**Authors:** Mazen Alazem, Na-Sheng Lin

**Affiliations:** Institute of Plant and Microbial Biology, Academia Sinica, Taipei, Taiwan

**Keywords:** abscisic acid, plant-virus interactions, defense responses, BaMV

## Abstract

Abscisic acid (ABA) is a key hormone involved in tuning responses to several abiotic stresses and also has remarkable impacts on plant defense against various pathogens. The roles of ABA in plant defense against bacteria and fungi are multifaceted, inducing or reducing defense responses depending on its time of action. However, ABA induces different resistance mechanisms to viruses regardless of the induction time. Recent studies have linked ABA to the antiviral silencing pathway, which interferes with virus accumulation, and the micro RNA (miRNA) pathway through which ABA affects the maturation and stability of miRNAs. ABA also induces callose deposition at plasmodesmata, a mechanism that limits viral cell-to-cell movement. Bamboo mosaic virus (BaMV) is a member of the potexvirus group and is one of the most studied viruses in terms of the effects of ABA on its accumulation and resistance. In this review, we summarize how ABA interferes with the accumulation and movement of BaMV and other viruses. We also highlight aspects of ABA that may have an effect on other types of resistance and that require further investigation.

## Introduction

Plants adapt to or tolerate stress through production of specific hormones that are produced at very low concentrations. One of the classical and well-studied phytohormones is abscisic acid (ABA), the importance of which is highlighted by its various roles in development (such as seed dormancy, germination, and floral induction) and stress responses (such as drought, salinity, and pathogen infection) (Mauch-Mani and Mauch, 2005; Wasilewska et al., 2008; Finkelstein, 2013; Humplik et al., 2017).

Abscisic acid affects the plant defense response to pathogens of different lifestyles, such as biotrophs that thrive on a living host without killing it and necrotrophs that cause host death and thrive on dead matter (Mauch-Mani and Mauch, 2005; Fan et al., 2009; Xu et al., 2013). However, the effects of ABA are multifaceted, depending on the pathosystem studied and the timing of induction (Ton et al., 2009). ABA can enhance plant defense if it is triggered at early stages of infection by closing stomata and inducing callose deposition at cell walls (Ton et al., 2009; Ellinger et al., 2013). In contrast, if a pathogen is successfully established inside a plant tissue, then ABA induction can hamper plant defense by antagonizing other hormone pathways such as those responsible for salicylic acid (SA) or ethylene synthesis (Anderson et al., 2004; Yasuda et al., 2008).

While ABA can both induce and reduce plant defense against fungal and bacterial pathogens, it appears to only enhance plant antiviral defense as shown for several viruses (Chen et al., 2013; Alazem et al., 2014; Alazem and Lin, 2015). Two ABA-dependent defense mechanisms against viruses have been reported in plants, callose deposition at plasmodesmata (PD)(Iglesias and Meins, 2000; De Storme and Geelen, 2014) and the RNA silencing pathway (Alazem and Lin, 2015; Alazem et al., 2017). In addition, ABA-related recessive resistance has been reported for two RNA viruses, bamboo mosaic virus (BaMV) and cucumber mosaic virus (CMV) (Alazem et al., 2014). These findings have attributed novel antiviral roles to ABA in plants, and have raised outstanding questions discussed below that require further investigations.

Bamboo mosaic virus is a positive-sense, single-stranded RNA virus of the *Potexvirus* genus (Family *Alphaflexiviridae*) with a genomic RNA of 6.4 Kb (Lin et al., 1994). BaMV genome encodes five open reading frames that translate into a replicase composed of three domains (a capping enzyme domain, a helicase-like domain, and an RNA-dependent RNA polymerase domain) (Li et al., 1998, 2001a,b; Huang et al., 2004), three movement proteins (Lin et al., 1994, 2004, 2006), and a capsid protein (Lan et al., 2010).

Since ABA effects on plant antiviral defense have been mostly studied using BaMV, here we summarize how ABA interferes with the accumulation, movement, and symptom development of BaMV and other viruses following infection. We also highlight several aspects of the ABA signaling pathway that may have potential effects on other types of antiviral resistance and that require further investigation.

## Virus Infection Induces ABA

Several RNA viruses have been shown to induce drought tolerance in plants, a phenomenon observed following infection by CMV, tobacco mosaic virus (TMV) and tobacco rattle virus (TRV) in different host plants including *Nicotiana tabacum*, *Beta vulgaris*, and *Oryza sativa* (Xu et al., 2008). Xu et al. (2008) ascribed this drought tolerance to the increase in the concentrations of osmoprotectants and antioxidants following viral infection. However, apart from the effects of osmolytes, drought tolerance is usually attributed to the increase of ABA content (Finkelstein, 2013). In fact, the increase of ABA content in virus-infected hosts has been reported for a number of compatible interactions (successful infection leading to disease) such as CMV/*Nicotiana benthamiana* (Alazem et al., 2014), BaMV/*Arabidopsis thaliana* and BaMV/*N. benthamiana* (Alazem et al., 2014), and TMV/*N. tabacum* (Fraser and Whenham, 1989). However, in some incompatible interactions (successful plant defense), viral infection does not induce ABA (Kovac et al., 2009; Baebler et al., 2014). For example, infection by potato virus Y (PVY^NTN^) of the resistant potato cultivar Sante, which harbors the Ry_sto_ extreme resistance gene, did not induce ABA. Instead, jasmonic acid (JA) increased within the first few hours after PVY^NTN^ infection (Flis et al., 2005; Kovac et al., 2009). Unaltered ABA content has also been reported for the resistant potato cultivar Rywal (carrying the *R*-gene *Ny-1*) following PVY infection, and for a resistant tomato cultivar (carrying the *R*-gene *Tm-1*) infected with TMV, although, in this latter case, the tomato cultivar resistant to TMV contained more ABA than a susceptible cultivar (Whenham et al., 1986; Baebler et al., 2014). Another study has shown that infecting resistant soybean (carrying the *R*-gene Rsv3) with an avirulent strain (G5H) of soybean mosaic virus (SMV) resulted in higher ABA content during the first 24 h of infection. Interestingly, SA was not induced throughout the time course of the experiment, but was increased late in response to a virulent SMV strain (G7H) (Seo et al., 2014).

Although viroids represent an interesting class of infectious entities without encoded proteins, studies on defense responses to viroids are still preliminary and lack solid conclusions on the roles of ABA or other hormones. For example, in response to potato spindle tuber viroid (PSTVd) infection (RG1 severe strain), ABA-related genes have shown different patterns of expression in tomato cultivars. Some genes in the ABA biosynthesis pathway were upregulated, such as the subunit of farnesyl transferase and the phospholipase D α-1, whereas few components of the guard cell ABA signaling pathway were downregulated (Owens et al., 2012). A similar study showed that no ABA or SA genes were induced following infection with the PSTVd RG1 strain, but only β*-1,3-glucanase* was induced at 25 days post-infection (Itaya et al., 2002). The difference between these two studies may be attributable to annotation of the tomato genome, which was not available at the time of the latter study. However, given the documented effect of ABA on callose accumulation, it can be speculated that ABA contributes to defense against viroids through callose. We will discuss the example of chrysanthemum stunt viroid (CSVd) spread in apical domains in the following section.

Since SA plays a major role in R-gene-mediated resistance, it is taken for granted that SA levels are elevated following viral infections (Baebler et al., 2014; de Ronde et al., 2014). However, there are some cases where JA or ABA are increased during early responses, such as of PVY^NTN^ or SMV (Kovac et al., 2009; Baebler et al., 2014; Seo et al., 2014). In both examples, SA was induced at later stages of infection. This concurrent induction of ABA/JA then SA suggests that each hormone contributes differently to defense. It remains unanswered why hormone responses in incompatible interactions differ according to the infecting virus.

Abscisic acid deficiency has been reported to have an influential role in *R*-gene-mediated resistance against bacterial pathogens. For example, high temperature inhibits nuclear localization of the proteins SNC1 and RSP4, which is required for resistance against the bacterial pathogen *Pseudomonas syringae*. However, when the ABA biosynthesis pathway was impaired, nuclear localization of both proteins was enhanced regardless of temperature, leading to temperature-insensitive resistance against *P. syringae* (Mang et al., 2012). Since the effect of ABA was achieved through the biosynthesis pathway (by testing *aba1* and *aba2* mutants) rather than through ABA signaling (by testing *abi1-1* and *abi4-1* mutants), the authors suggested a role for *ABA2* in *R*-gene-mediated resistance (Mang et al., 2012). Similar effects of ABA on *R*-genes that function against viruses are possible. Some *R*-genes have previously been shown to be temperature-sensitive, such as the *Rx*-gene against potato virus X (PVX) and the *N*-gene against TMV, but when plant culture temperatures were increased from 22 to 28°C the hypersensitive response disappeared in infected tobacco and tomato plants (Samuel, 1931; Whitham et al., 1996; Wang et al., 2009). A recent study revealed that the temperature-sensitive *Wsm1* gene, which confers resistance to wheat streak mosaic virus (WSMV) and triticum mosaic virus (TriMV), and *Wsm2* that confers resistance to WSMV alone, block the systemic movement of both viruses in wheat at low temperature. Both viruses failed to enter the leaf sheaths of inoculated leaves at 18°C (but not at 24°C), thereby conferring resistance by impairing their long-distance movement (Tatineni et al., 2016). Whether or not ABA mediates these effects has yet to be investigated.

## ABA-Dependent Callose Accumulation Is An Antiviral Mechanism

Plant viruses move from cell to cell via PD, with specific viral proteins (mostly movement proteins) modifying PD and increasing the size exclusion limit (which determines the size of the molecules traversing PD), thereby allowing the large viral movement complex to pass through (Fridborg et al., 2003; Lucas, 2006; Su et al., 2010; Heinlein, 2015). Trafficking through PD can be modulated by the controlled deposition of callose, a polysaccharide of the class β-1,3-glucan, at the necks of PD (Iglesias and Meins, 2000; Li et al., 2012b). Callose is a key component involved in cell fortification, and is found in different tissues at various developmental stages because it is required for growth and development. It is encoded by *callose synthase* (*CalS*) genes (or *glucan synthase-like* [*gsl*]), a gene family comprising 12 members in *Arabidopsis* that are involved in producing callose in different tissues/organelles (Verma and Hong, 2001; Dong et al., 2008; Ellinger and Voigt, 2014). Callose is also involved in plant response to biotic stress, with its deposition on the cell wall and at PD being important for restricting pathogen progression (Mauch-Mani and Mauch, 2005; Luna et al., 2011; Ellinger and Voigt, 2014). Among *CalS* genes, *CalS10* (or *GSL8*) has been identified as the primary regulator of callose deposition at PD (Guseman et al., 2010; Ellinger and Voigt, 2014; Han et al., 2014).

Plants control callose levels by the action of β-1,3-glucanases, which are hydrolytic enzymes that catalyze cleavage the 1,3-β-D-glucosidic linkages into single β-1,3-glucan units (Doxey et al., 2007; De Storme and Geelen, 2014). β-1,3-glucanases are a diverse group of enzymes of different sizes, structure and localization (Doxey et al., 2007), and genes encoding these enzymes (e.g., PR2) are known to be induced during viral infections, which result in the removal of callose and thereby facilitates viral trafficking (Rezzonico et al., 1998; Kitajima and Sato, 1999; Oide et al., 2013).

In contrast, ABA has been shown to suppress expression of PR2, which allows more callose to accumulate at PD (Rezzonico et al., 1998) and thereby reduces viral intercellular movement and spread (Iglesias and Meins, 2000; Heinlein, 2015). The negative effect of ABA on β-1,3 glucanases suggests that ABA can increase callose accumulation in different tissues and organelles (PD, cell wall, phloem sieve plates). In fact, few studies listed below have shown the link between ABA induction, callose deposition and restriction of virus movement.

Below, we summarize the findings on the roles of callose in both compatible and incompatible plant–virus interactions:

### Roles of Callose in Compatible Interactions

Most of the cases reporting a role for ABA in plant defense against viruses involve compatible interactions. ABA pretreatment has been shown to reduce levels of different RNA viruses, such as tobacco necrosis virus (TNV) on *Phaseolus vulgaris* (Iriti and Faoro, 2008), TMV on *N. tabacum* (Whenham et al., 1986; Fraser and Whenham, 1989), and BaMV on *A. thaliana* (Alazem et al., 2014). These works postulated that enhanced callose deposition at PD could explain the ABA-dependent resistance, which is supported by the inability of TNV, for example, to spread in ABA-treated leaves (Iriti and Faoro, 2008).

In compatible interactions, the response of plants to virus or viroid infections is not strong enough to prevent spread of the viral agents to other tissues, which is evident from the levels of defense responses such as ABA, SA, callose and reactive oxygen species (ROS) (Kovac et al., 2009; Baebler et al., 2014; Seo et al., 2014; Lopez-Gresa et al., 2016). Considering that the biosynthesis pathway of ABA (like other hormones such as SA and JA) takes place in the chloroplast (Finkelstein, 2013), and that certain viruses and viroids interfere with several machineries in such plastids (Zhao et al., 2016), this might be the reason why some plants do not produce sufficient amounts of ABA or callose in response to infection in leaves. In contrast, callose deposition in meristemic tissues seems to be more efficient in preventing viroid spread. For instance, the response of two different Argyranthemum cultivars (Yellow Empire and Border Dark Red) to infection with chrysanthemum stunt viroid (CSVd) revealed that less callose was deposited at PD in the shoot apical meristem (SAM) of Yellow Empire compared to Border Dark Red, which resulted in the spread of CSVd to the uppermost cell layers in the apical dome and the youngest leaf primordia 1 and 2 of Yellow Empire (Zhang Z. et al., 2015). However, the SAM in the Border Dark Red cultivar presented more callose particles, which prevented CSVd from spreading beyond the lower part of the apical domain and elder leaf primordia (Zhang Z. et al., 2015). Which factor controls or induces callose deposition in SAM is unknown. Notably, both cultivars showed disease symptoms after infection with CSVd, which raises the question of whether callose deposition at PD occurs in other tissues (such as leaves) and whether this accumulation affects CSVd movement (Flores, 2016).

### Roles of Callose in Incompatible Interactions

Callose deposition has been documented in resistant soybean plants (carrying the *R*-resistance gene) in response to SMV. This response restricted SMV to the inoculated sites as no SMV RNA was detected beyond these sites (Li et al., 2012b). The same study also showed that susceptible soybean plants infected with SMV could not accumulate callose and, as a result, SMV infection spread (Li et al., 2012b). A similar study showed that another soybean cultivar that possessed the *Rsv3* gene exhibited extreme resistance to SMV (Seo et al., 2014). This resistance was achieved by a subset of *PP2C*-encoding genes that comprise components of the ABA signaling pathway and that are induced by ABA. Recognition of SMV’s cylindrical inclusion effector by the cultivar’s Rsv3 protein induced the ABA pathway and activated the *PP2Ca3* gene which, in turn, induced callose deposition and conferred extreme resistance against SMV (Seo et al., 2014). However, the mechanism linking PP2C proteins and *CalS* genes (or their protein products) or β-1,3-glucanases is unknown. Thus, induction of ABA in some incompatible plant–virus interactions suggests a role for ABA in innate immunity that needs to be experimentally validated (Whenham et al., 1986; Melotto et al., 2008; Kovac et al., 2009; Pacheco et al., 2012; Seo et al., 2014).

### Callose in the Early Antiviral Response: Is it Controlled by SA or ABA?

**FIGURE 1:**
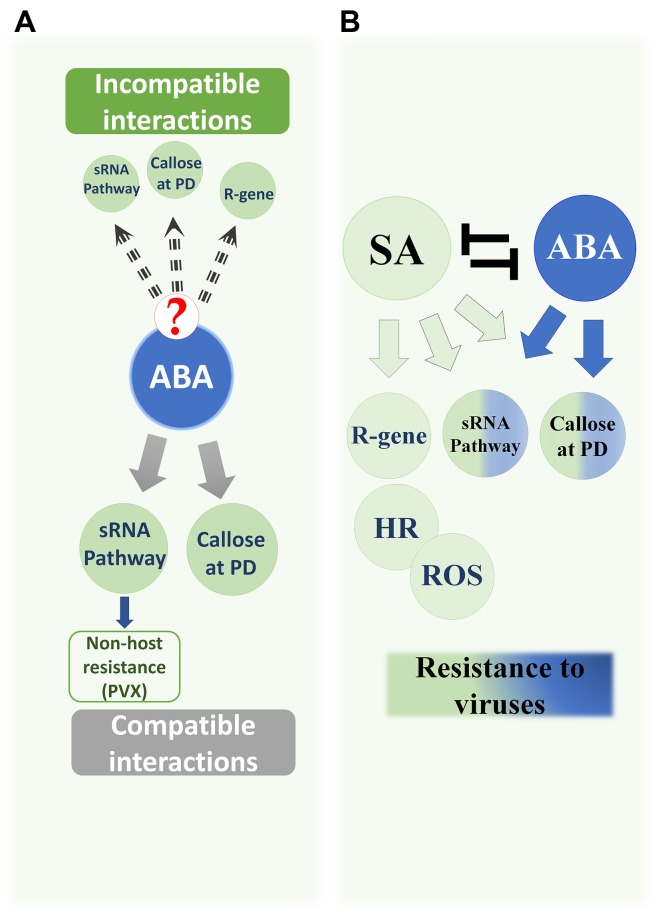
Antiviral Roles of ABA and SA in plants. **(A)** ABA’s documented roles against viruses in compatible interactions: (i) Enhanced callose deposition at plasmodesmata (PD). (ii) Positive regulation of several AGO genes in the sRNA pathway, which reduces BaMV and PVX levels. ABA has an additional role in non-host resistance against PVX because ABA deficiency resulted in limited accumulation of AGO2 so that *Arabidopsis* became susceptible to PVX accumulation and systemic movement. The role of ABA in incompatible interactions has not been addressed. However, the effects of ABA on the callose and sRNA pathway, as well as the increased ABA content in some incompatible interactions, may suggest a role in such interactions. **(B)** The antagonistic pathways SA and ABA positively regulate common subsets of antiviral resistance mechanisms: callose deposition and sRNA (half-green half-blue circles). SA controls *R*-gene resistance, induces hypersensitive responses (HR), and the accumulation of reactive oxygen species (ROS) (green circles). SA contributes to the production of siRNAs and enhances callose deposition during early immune responses in incompatible interactions. In addition, exogenous application of SA increases plant tolerance to viruses in compatible interactions, which is supported by the increased susceptibility in lines with an impaired SA pathway.

Early induction of ABA in some incompatible interactions supports the hypothesis that ABA plays a role during early immune responses against some viruses (Whenham et al., 1986; Melotto et al., 2008; Pacheco et al., 2012; Seo et al., 2014). However, it remains unclear whether or not callose deposition at that stage is completely ABA-dependent because no ABA mutants have been assessed to confirm the role of ABA-dependent callose deposition in incompatible interactions (**Figure 1A**).

In contrast, much more is known about how SA affects PD and callose. Several reports have shown that SA induces PD closure and impairs their permeability by increasing the amount of callose deposited at PD (Wang et al., 2013; Cui and Lee, 2016). This effect requires the action of plasmodesmata-located protein 5 (PDLP5), which is dependent on NPR-1 (Wang et al., 2013). PDLP5 controls the expression of *CalS1* and *CalS8* genes that are responsible for callose synthesis and deposition at PD in response to SA treatment (Cui and Lee, 2016). The major gene involved in callose deposition at PD, *CalS10*, functions independently of PDLP5 or SA, as evidenced by the normal plasmodesmal permeability induced by exogenous SA in the *cals10-1* mutant (Cui and Lee, 2016).

Despite the fact that both SA and ABA enhance callose deposition at PD (**Figure 1B**), the mechanism regulating this effect is quite different in each case. While the action of SA is mediated directly via specific genes (*PDLP5*, *CalS1*, and *CalS8*), ABA exerts a general indirect effect by transcriptionally decreasing β-1,3-glucanases that proteins may target all kinds of callose (Oide et al., 2013; Wang et al., 2013; Cui and Lee, 2016).

It is important to note that, in some cases, ABA does not lead to more callose deposition and, depending on growth conditions, its effect on callose deposition can even be reversed. For example, under conditions of low light intensity, high sucrose levels and the addition of Gamborg’s vitamin to growth medium, applications of ABA have been shown to repress callose deposition (Luna et al., 2011).

### Suppression of Callose-Mediated Defense

Although ABA reduces the expression of β-1,3-glucanases, which are responsible for callose degradation, some viroids have evolved different ways to overcome the potential increase in callose deposition at PD. For example, PSTVd in tomato benefits from the activation of the small RNA (sRNA) pathway that produces sRNAs derived from the virulence modulating region of PSTVd. These viroid-derived sRNAs target the *CalS11-like* and *CalS12-like* genes to interfere with callose synthase mRNA levels. However, their roles in callose accumulation at PD in tomato plants is unknown (Adkar-Purushothama et al., 2015a). In addition, some viruses recruit host factors that help degrade or remove callose from PD such as TAG4.4/SAG2.3, AtBG_ppap (a beta-glucanase), ANK/TIP1-3, or others, so that callose does not hinder viral intercellular trafficking (Burch-Smith and Zambryski, 2016).

Several gaps remain in our knowledge of the roles of the antagonistic ABA and SA pathways in callose-mediated restriction of virus spread in incompatible interactions. Some studies addressed the roles of either hormone in incompatible interactions and callose deposition (**Figure 1B**). However, since both hormones appear to affect callose levels, a study that jointly tests the effects of both hormones on β*-1,3-glucanase* and *CalS* genes and proteins, and consequent callose accumulation at PD or cell walls, would greatly clarify how cells react early to infection and induce defense responses.

## ABA-Dependent Antiviral Defense Through RNA Silencing Pathways

Because sRNAs are repressors of gene expression, their mechanism of action is referred as RNA silencing, gene silencing, or RNA interference (Vaucheret, 2006). RNA silencing occurs on two levels; transcriptional gene silencing, and RNA degradation [or post-transcriptional gene silencing (PTGS)], which correlates with the accumulation of short-interference small RNAs (siRNAs) (Vaucheret, 2006). siRNAs are loaded into the RNA-Induced Silencing Complex (RISC) and guide Argonaute (AGO) proteins (the key player in RISC) to cleave or inactivate RNAs derived from transposons, viral-, trans-, or endogenous- genes leading to their degradation (Vaucheret, 2006; Chapman and Carrington, 2007). In *Arabidopsis*, the backbone of the RNA silencing pathway consists of proteins from three families: (1) The Dicer-Like (DCL) family, which comprises four genes (*DCL1*, *DCL2*, *DCL3*, and *DCL4*). (2) The AGO family, which comprises of 10 functional members (From *AGO1* to *AGO10*, with a pseudo *AGO8*) (Takeda et al., 2008; Mallory and Vaucheret, 2010; Seo et al., 2013). (3) The RNA-directed RNA polymerase (RDR)s family, which comprises three functional genes; *RDR1*, *RDR2*, and *RDR6*. The antiviral RNA silencing pathway is PTGS-based, and several genes in the DCL, AGO, and RDR families appear to have redundancy in their function against invading viruses (Vaucheret, 2008; Garcia-Ruiz et al., 2010, 2015; Pelaez and Sanchez, 2013; Seo et al., 2013). While siRNAs, which are derived from viruses, transgenes or from a subset of endogenous genes, are *cis*-acting siRNAs and therefore their action is termed as autosilencing, micro-RNAs (miRNA) originate from distinct genes, different from the ones they regulate, with their action referred to as heterosilencing (Bartel, 2004; Vaucheret, 2006). Viruses have evolved viral suppressors for RNA silencing (VSR) that enable them to counteract the antiviral RNA silencing pathway (Li and Ding, 2006; Burgyan and Havelda, 2011). Generally, VSR are multifunctional and play vital roles is viruses’ movement, replication or pathogenesis (Cao et al., 2010; Csorba et al., 2015). For example, the movement protein “triple gene block protein 1” in several potexviruses has VSR function along with its role in virus movement (Senshu et al., 2009; Lim et al., 2010; Brosseau and Moffett, 2015). Viruses often encode one VSR that can interfere the RNA silencing pathway at different steps such as binding dsRNA, preventing siRNA translocation or RISC assembly, or interacting with AGO proteins and impairing their silencing function (Li and Ding, 2006; Jin and Zhu, 2010; Burgyan and Havelda, 2011; Kenesi et al., 2017).

Until very recently, ABA-dependent callose deposition at PD was the only documented link between ABA and resistance to viruses. However, a recently revealed connection between ABA and the RNA silencing pathway has added another role for ABA in resistance to viruses (Alazem and Lin, 2015; Alazem et al., 2017). ABA-dependent defense against BaMV and PVX in *Arabidopsis*, for example, is mainly achieved through the RNA silencing pathway, not through callose deposition at PD (Jaubert et al., 2011; Alazem et al., 2017).

### Role of ABA in Endogenous sRNA Pathways

Expanding evidence has attributed a regulatory role for ABA in sRNA pathways, such as the siRNA and miRNA pathways. Previous works reported that ABA is required for stabilization of Cap binding proteins (CBP) 20 and 80 in a post-translational mechanism (Kim et al., 2008). These two proteins function in the formation of pre-miRNA transcripts and facilitate splicing during miRNA biogenesis. In addition, *cbp20* and *cbp80* mutants render plants hypersensitive to ABA. It is known that CBP20 is a negative regulator of ABA-dependent drought tolerance, and mutation of this gene renders plants tolerant to drought (Papp et al., 2004; Kim et al., 2008; Kuhn et al., 2008). Similarly, *CBP80* downregulation in potato reduced miR159 levels, thereby allowing accumulation of the miR159-target genes *MYB33* and *101* and consequently increasing drought tolerance (Pieczynski et al., 2013). In fact, mutants of several components of the miRNA pathway such as hyponastic leaves 1 (*HYL1*), HUA enhancer 1 (*HEN1*) or *DCL1* also exhibit hypersensitivity to ABA (Lu and Fedoroff, 2000; Zhang et al., 2008). Other mutants have shown ABA supersensitivity such as *dcl2*, *dcl3*, *dcl4* and their corresponding triple mutant. Expression of ABA-responsive genes such as *RD22* and *ABF3* was significantly increased in all *dcl* mutants. The mutants *dcl2*, *dcl3* and *dcl4*, but not *dcl1*, showed increased levels of *ABI3, ABI4*, and *ABI5* gene products (Zhang et al., 2008). Of note, *abi3-1* and *abi4-1* increased plant susceptibility to BaMV infection, but the genes regulated by these factors are still unknown (Alazem et al., 2014). Actually, several works have indicated that abiotic stresses such as drought, salinity, or cold stress (all of which are partially regulated by ABA) induce genes in the DCL and RDR families in tomato, maize and *Populus trichocarpa* (Qian et al., 2011; Bai et al., 2012; Zhao et al., 2015). The direct effect of ABA on the expression of DCLs or RDR is exemplified by the increased expression of *RDR1* in *A. thaliana* and of all *RDRs* in *O. sativa*, but only RDR6 was responsible for persistent ABA post-transcriptional control of gene silencing in *O. sativa* (Yang et al., 2008; Hunter et al., 2013).

### Antiviral Role of ABA through Regulation of AGOs

Argonaute proteins are integral players in all sRNA pathways in plants and animals, comprising a family of 10 members in *Arabidopsis* (Carbonell, 2017). By associating with different sRNAs, they regulate the expression of many genes and thereby control several aspects of growth, development and resistance to viruses (Vaucheret, 2008; Carbonell and Carrington, 2015; Zhang H. et al., 2015). All AGOs have been reported to reduce levels of different viruses, with variations in efficiency probably due to the effects of VSRs (Brosseau and Moffett, 2015; Carbonell and Carrington, 2015; Brosseau et al., 2016; Alazem et al., 2017). For example, when deleting the VSR of PVX (ΔP25), all overexpressed AGOs downregulated PVX-ΔP25 in *N. benthamiana* (Brosseau and Moffett, 2015). miR168 levels, which maintains *AGO1* homeostasis, are regulated by ABA (Li et al., 2012a). Li et al. (2012a) found that *AGO1* RNA is not induced within 12 h of ABA treatment in *Arabidopsis* seedlings because of the effect of miR168, but another study found that extending the effect of ABA to 4 days induced not only *AGO1* but also *AGO2* and *AGO3* (Alazem et al., 2017). The latter study conducted experiments on ∼30 day-old *Arabidopsis*, compared to the 7-day old seedlings used by Li et al. (2012a). These results were confirmed in ABA-deficient mutants (*aba2-1* and *aao3*), showing that *AGO1*, *AGO2*, and *AGO3* were expressed at very low levels (Alazem et al., 2017). In that study, BaMV infection also induced *AGO1*, *2* and *3* expression, but when *aba2-1*- and *aao3*-deficient mutants were infected with BaMV, *AGO1* and *2* but not *3* failed to accumulate to wild-type levels, indicating the expression of these AGOs is ABA-dependent. Furthermore, ABA was found to have negative effects on *AGO4* and *AGO10* expression, but differential effects on *AGO7* expression, since ABA treatment did not induce AGO7 mRNA accumulation in wild-type plants but ABA-deficient mutants (*aba2-1 and aao3*) showed significantly reduced expression of AGO7 (Alazem et al., 2017). These findings imply that ABA generally affects several genes in the RNA silencing pathway, perhaps representing an important tool by which ABA tunes plant responses to different stimuli.

Although AGO1 has antiviral activity against several viruses (Morel et al., 2002; Qu et al., 2008), BaMV levels were not reduced in the *4mAGO1* transgenic line in which *AGO1* was made resistant to the downregulatory effect of the AGO1–miR168 complex by four mismatches that prevent binding with miR168a. In the same context, the miR168a-2 mutant accumulates the AGO1 protein, but BaMV levels were still unaffected in this mutant compared to wild-type plants (Vaucheret, 2009; Alazem et al., 2017). It could be that either AGO1 has no clear effect against BaMV or that a VSR of BaMV (probably TGBp1) impairs the antiviral activity of AGO1. In contrast, the *ago1-27* mutant showed reduced BaMV levels compared to wild-type plants because of the increased expression of *AGO2* and *AGO3* levels in this mutant. Surprisingly, BaMV levels were not affected in the ABA-treated *ago3-2* mutant and were not significantly reduced in the ABA-treated *ago2-1* mutant compared with corresponding mock-treated mutant (Alazem et al., 2017). These findings imply that ABA-dependent resistance to BaMV is mainly achieved through AGO2 and AGO3, and that callose deposition at PD may not be the main resistance mechanism controlled by ABA, at least in some compatible interactions. In fact, restriction of viruses to the sites of infection during incompatible interactions can also be ascribed to the activity of the RNA silencing pathway, and further studies on this topic could reveal much about the involvement of ABA in incompatible interactions. In the same context, it was found that the RNA silencing pathway controls the non-host resistance of *A. thaliana* to PVX infection, mainly through AGO2 (Jaubert et al., 2011). This finding was also confirmed for the *aba2-1* mutant, which produces very little AGO2, thereby allowing PVX to accumulate locally and move systemically compared to the scenario in wild-type plants (Alazem et al., 2017).

Several studies have addressed the roles of the RNA silencing pathway in resistance to viroids since RNA or DNA replication intermediates trigger this pathway (Minoia et al., 2014; Adkar-Purushothama et al., 2015b; Carbonell and Daros, 2017). Since ABA regulates several genes in this pathway, ABA could also play a role in mediating resistance to viroids. For example, Minoia et al. (2014) found that *A. thaliana* AGO1, AGO2, AGO2, AGO3, AGO4, AGO5, and AGO9 were loaded with PSTVd-derived sRNA in infected *N. benthamiana* plants. Given the regulatory role of ABA in *AGO1*, *2*, and *3*, it is possible that ABA may participate in resistance to viroids through these *AGOs*.

## ABA and Recessive Resistance

**FIGURE 2:**
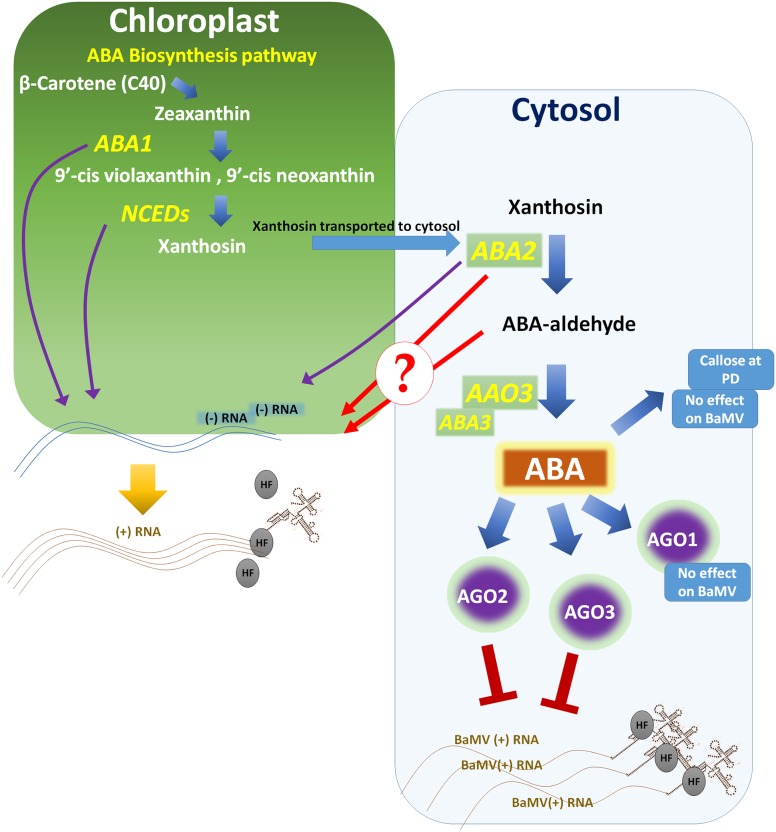
Abscisic acid (ABA) effects pathway on BaMV accumulation and plant antiviral resistance. A large part of ABA biosynthesis takes place in the chloroplast. Impairment of genes that function in the chloroplast, such as *ABA1* in *Nicotiana benthamiana* or *NCED3* in *Arabidopsis thaliana*, significantly reduces BaMV levels. The last two steps in ABA biosynthesis take place in the cytosol, where ABA2 coverts xanthosin into ABA-aldehyde and AAO3 reduces ABA-aldehyde to produce ABA. *ABA2* mutants have markedly reduced levels of BaMV (-)RNA. Whether ABA2, ABA-aldehyde or other factors controlled by ABA2 are required for BaMV to accumulate is unknown. In contrast, mutation of *AAO3* and downstream genes increases susceptibility to BaMV. ABA partially controls the expression of the AGO gene family and induces *AGO1*, *2* and *3*, with AGO2 and 3 but not AGO1 acting against BaMV. In addition, ABA induction of callose is ineffective against BaMV because plants silenced in *CalS10* still show resistance after ABA treatment. HF, host factor.

Recessive resistance is defined as the loss of susceptibility when an important host factor required for virus replication is impaired (Hashimoto et al., 2016). To date, most of the discovered recessive-resistance genes belong to the translation initiation factor (eIF) 4E and eIF4G groups (Hashimoto et al., 2016). However, other host factors are involved in BaMV accumulation and they localize to the cytosol and chloroplast (**Figure 2**). Further information on those factors is described in a recent review (Huang et al., 2017). Here, we briefly focus on the chloroplast-related genes since ABA and other hormones are biosynthesized in chloroplasts.

Chloroplast phosphoglycerate kinase (cPGK) interacts with the 3′-untranslated region of BaMV to direct BaMV RNA to the chloroplasts, and silencing or mislocalization of cPGK significantly reduces BaMV levels (Lin et al., 2007; Cheng et al., 2013). BaMV Minus-strand (-) RNA has been detected within chloroplasts, which suggests localization of BaMV replication intermediates there (Lin et al., 2007; Cheng et al., 2013). In accordance with these findings, the *Arabidopsis* genotype *Cvi-0* comprises a natural recessive resistance gene, *rwm1*, which encodes a mutated cPGK protein and confers resistance to two potyviruses (watermelon mosaic virus and plum pox virus) but not to the potexvirus PVX or the cucumovirus CMV (Lin et al., 2007; Ouibrahim et al., 2014; Poque et al., 2015). Furthermore, the ABA biosynthesis gene *ABA2* and the upstream gene *NCED3* are important for BaMV (-)RNA accumulation (Alazem et al., 2014). Because of the feedback loop in the ABA biosynthesis pathway, the *nced3* mutant exhibited low levels of ABA2, accounting for the low level of BaMV in that mutant. Hence, ABA2 is required for a step preceding BaMV translation, and a similar role was also suggested for the accumulation of CMV in *A. thaliana* (Alazem et al., 2014).

In the same context, in the ABA biosynthesis pathway, ABA1 and NCED3 are localized in the chloroplasts, whereas ABA2 and AAO3 (the *aao3* mutant is highly susceptibility to BaMV unlike the *aba2-1* mutant; Alazem et al., 2014) are localized in the cytosol. Hence, the ABA biosynthesis pathway in the chloroplasts may be required for BaMV accumulation (**Figure 2**). It is still not known whether this recessive resistance is the result of *ABA2* substrate or other factors controlled by *ABA2*. The different localization of cPGK and ABA2 (Cheng et al., 2002) and the different nature of the substrates handled by them may suggest different roles.

## Concluding Remarks

The increased expression of several genes of the *AGO*, *RDR*, and *DCL* families in response to ABA, as well as the observation that several of these genes are important players in the antiviral RNA silencing pathway, strengthens the notion that the antiviral role of ABA is partially achieved through the RNA silencing pathway. The additional effect of ABA-dependent callose deposition at PD thus endows ABA with a dual function in restricting virus spread (**Figure 1A**). Both mechanisms have been assessed only for BaMV (**Figure 2**), and the findings have shown that callose deposition is not the only defense mechanism mediated by ABA. Further studies with other viruses and viroids will reveal how efficient these mechanisms are in different pathosystems.

The antagonism between SA and ABA is well-documented, whereby downstream genes of either pathway are suppressed if the other hormone is applied or induced (Yasuda et al., 2008; Zabala et al., 2009; Moeder et al., 2010). It is known that viruses disrupt hormonal balance in compatible interactions, leading to simultaneous induction of some antagonistic pathways such as ABA and SA in the case of BaMV and CMV (Alazem et al., 2014). However, because of the positive effects that both hormones have on the same subset of defense responses (**Figure 1B**), it is not clear whether these two antagonistic pathways actually act antagonistically during viral infections. Antagonism is evident in some incompatible interactions in which the induction of these pathways is strong, sequential and not concurrent, implying that each hormone takes a role in triggering several redundant antiviral mechanisms (Alazem and Lin, 2015), but experimental evidence is lacking.

## Author Contributions

MA and N-SL wrote, revised, and approved this manuscript.

## Conflict of Interest Statement

The authors declare that the research was conducted in the absence of any commercial or financial relationships that could be construed as a potential conflict of interest. The reviewer AC and handling Editor declared their shared affiliation.
